# Green Nanotechnology: Advancement in Phytoformulation Research

**DOI:** 10.3390/medicines6010039

**Published:** 2019-03-14

**Authors:** Ajay Verma, Surya P. Gautam, Kuldeep K. Bansal, Neeraj Prabhakar, Jessica M. Rosenholm

**Affiliations:** 1 Pharmaceutical Sciences Laboratory, Faculty of Science and Engineering, Åbo Akademi University, 20520 Turku, Finland; kbansal@abo.fi (K.K.B.); nprabhak@abo.fi (N.P.); jerosenh@abo.fi (J.M.R.); 2 CT Institute of Pharmaceutical Sciences, Jalandhar 144020, India; suryagautam@ymail.com

**Keywords:** green nanotechnology, phytoformulations, green engineering, gold (Au) nanoparticles, nanoproducts

## Abstract

The ultimate goal of any scientific development is to increase well-being and human health. Novel strategies are required for the achievement of safe and effective therapeutic treatments beyond the conventional ones, and society needs new requirements for new technologies, moving towards clean and green technology development. Green nanotechnology is a branch of green technology that utilizes the concepts of green chemistry and green engineering. It reduces the use of energy and fuel by using less material and renewable inputs wherever possible. Green nanotechnology, in phytoformulations, significantly contributes to environmental sustainability through the production of nanomaterials and nanoproducts, without causing harm to human health or the environment. The rationale behind the utilization of plants in nanoparticle formulations is that they are easily available and possess a broad variability of metabolites, such as vitamins, antioxidants, and nucleotides. For instance, gold (Au) nanoparticles have attracted substantial attention for their controllable size, shape, and surface properties. A variety of copper (Cu) and copper oxide (CuO) nanoparticles have also been synthesized from plant extracts. Titanium dioxide and zinc oxide nanoparticles are also important metal oxide nanomaterials that have been synthesized from a number of plant extracts. International and domestic laws, government and private-party programs, regulations and policies are being carefully reviewed and revised to increase their utility and nurture these nanoscale materials for commercialization. Inspiring debates and government initiatives are required to promote the sustainable use of nanoscale products. In this review, we will discuss the potential of the utilization of plant extracts in the advancement of nanotechnology.

## 1. Introduction

**Figure 1:**
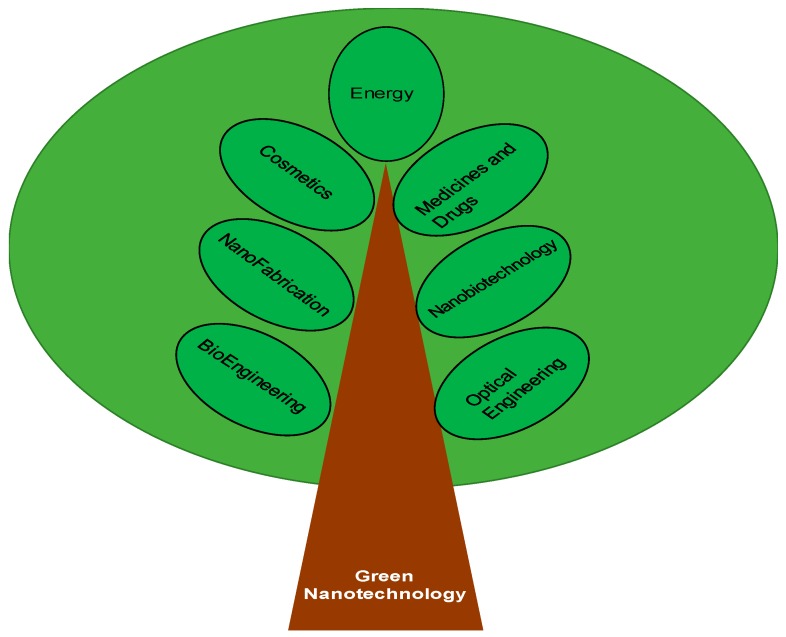
Branches of green nanotechnology.

Nanotechnology is cited as a key technology of the 21st century and has generated a great deal of excitement world-wide, but it has been slowed down because of the poor understanding of hazards associated with nanotechnology and fewer policies to manage new risks. Researchers, however, continue to move ahead, engaging themselves to conquer the challenges ranging from managing, producing, funding, regulatory, and technical aspects. Green nanotechnology is a branch of green technology that utilizes the concepts of green chemistry and green engineering, where the word “green” refers to the use of plant products (Figure 1). It reduces the use of energy and fuel by using less material and renewable inputs wherever possible. Furthermore, nanotechnological products, processes, and applications are expected to contribute significantly to environmental and climate protection by saving raw materials, energy, and water, as well as by reducing greenhouse gases and hazardous waste. Increased energy efficiency, reduced waste and greenhouse gas emission, and decreased consumption of non-renewable raw materials are the main advantages of green nanotechnology. Green nanotechnology offers a great opportunity to stop the adverse effects before they occur [1,2].

Green nanotechnology does not ascend de novo; rather, it forms on the principles of green chemistry and engineering. Apart from such obvious areas as the development of solar cells, biofuels, and fuel cells, green nanotechnology applications might involve the use of nanomaterials in clean production processes that synthesize nanoparticles, using sunlight or by recycling industrial waste products into nanomaterials. There is some “truly” green nanotechnology, i.e., fully growing nanomaterials in plants—however, they will never reach the scale required for the industrial production of nanomaterials. In order to make a conclusive observation, green nanotechnology needs a full process assessment like other industrially manufactured products [3,4].

## 2. Herbal Approach for Developing Nanoparticles

The activity of herbal medicines depends on the overall function of active components, as all the constituents provide synergistic action and, thus, improve the therapeutic value. Each active constituent is related to each other and they all play significant roles (Table 1). On the other hand, the insoluble character of most of the drugs of herbal origin leads to lower bioavailability and, because of this, systemic clearance is increased and frequent administration or a higher dose is required—all of which renders the drug a low-class drug for therapeutic use.

In phytoformulation research, developing nanotechnology-based dosage forms, e.g., solid lipid nanoparticles (SLNs), polymeric nanoparticles (nanospheres and nanocapsules), proliposomes, liposomes, nanoemulsions, etc., has a great number of advantages for herbal drugs. These include enhancement of solubility and bioavailability, improvement of stability, suppression of toxicity, improvement of pharmacological activity, sustained delivery, improving tissue macrophage circulation, and defense against physical and chemical degradation. Therefore, problems associated with plant medicines can be overcome with nano-sized drug delivery systems (NDDS) of herbal drugs, having a potential future for enhancing their activity. Hence, including nanocarriers as an NDDS in conventional medicine systems would be necessary to combat more chronic diseases like diabetes, cancer, asthma, and others, with the aid of herbal drugs [5,6,7].

## 3. Nanoparticles Synthesized from Plant Extracts

### 3.1. Gold and Silver Nanoparticles

Au nanoparticles have gained substantial attention due to their controllable size, shape, and surface properties [15]. Because of these unique properties, gold nanoparticles have been studied for potential applications in areas such as biosensors, hyperthermia therapy [16], antibacterial drugs, genetic engineering, and delivery platforms for therapeutics. Environmentally friendly sources of Au nanoparticles are achieved by employing plants, as they are biological factories via green chemistry-based techniques. The study of nanoparticle syntheses also discovered that a variety of shapes, including rod-shaped, irregular, decahedral, icosahedral, and hexagonal, could be produced, depending on the pH of the reaction medium. Furthermore, a leaf extract of eucalyptus macrocarpa could be used to synthesize gold nanoparticles (Table 2). The results from this study show that spherical particles with a size ranging from 20 to 80 nm were obtained as the main product [17].

In addition to synthesizing pure metal nanoparticles by plants in this way, several authors have also reported alloying Au and Ag to investigate the properties of the resulting bimetallic nanoparticles. This bimetallic nanoparticle synthesis comprises a competitive reduction process between two aqueous solutions, each of which contain a different metallic ion precursor that is reacted with a plant extract. In the case of bimetallic nanoparticles, gold has a larger reduction potential than silver, so gold will form first and create the core of the resulting core–shell structure. Subsequently, the reduction of Ag ions, in the same way, results in Ag coalescing on the core to form the shell. There are some plants that have been effectively used to synthesize bimetallic (Au-Ag) nanoparticles, including cashew nut, neem, and West Indies mahogany [29,30,31].

### 3.2. Copper and Copper Oxide Nanoparticles

A variety of copper (Cu) and copper oxide (CuO) nanoparticles have been synthesized from plant extracts. Cu nanoparticles of magnolia leaf extract have been biologically synthesized to develop stable nanoparticles sized 40 to 100 nm. Furthermore, the activity of Cu nanoparticles has shown potential antibacterial activity against cells of *Escherichia coli* [32]. *Syzygium aromaticum* (clove) extracts have been used in the synthesis of Cu nanoparticles with a spherical to granular morphology and a mean particle size of 40 nm [33]. Cu nanoparticles have been synthesized by using the stem latex of *Euphorbia nivulia,* that is, common milk hedge. These nanoparticles are stabilized by peptides and terpenoids that are present in latex. Furthermore, these nanoparticles are reported to be toxic to human adenocarcinomic alveolar basal epithelial cells [34].

### 3.3. Palladium and Platinium Nanoparticles

Satishkumar et al. [35] developed palladium nanoparticles from *C. zeylanicum* (cinnamon) bark extract. During synthesis, the temperature, concentration, and reaction pH of the bark extract changed, but morphology and particle size (15 to 20 nm) were not influenced. Using *Annona squamosa* (custard apple) peel extract, palladium nanoparticles were also synthesized, ranging in size from 75 to 85 nm [36]. *Camellia sinensis* (tea) and *Coffea arabica* (coffee) extracts have been used to synthesize palladium nanoparticles with sizes ranging from 20 to 60 nm and a cubic crystal symmetry in the center [37]. Song et al. [38] reported the first platinum nanoparticles of *Diospyros kaki* (persimmon) leaf extract, having sizes of 2 to 12 nm. Lately, particle size- and shape-controlled biological synthesis of platinum nanoparticles has also been reported. Plant wood for the nanometer scale has been used for this purpose [39]. For instance, Coccia et al. [40] reported isolated lignin from red pine (*Pinus resinosa*) for producing palladium and platinum nanoparticles.

### 3.4. Titanium Dioxide and Zinc Oxide Nanoparticles

These important metal oxide nanomaterials have been synthesized from a number of plant extracts. For instance, Roopan et al. [41] established that TiO_2_ nanoparticles could be effectively synthesized from *Annona squamosa* peel; meanwhile, from *Nyctanthes arbor-tristis* leaf extracts, round particles were found, ranging in size from 100 to 150 nm [42]. *Eclipta prostrata* leaf extracts could also produce particles, with a size range of 36 to 68 nm [43,44]. Velayutham et al. synthesized TiO_2_ nanoparticles using *Catharanthus roseus* leaf extract, ranging in size from 25 to 110 nm and irregularly shaped. The suspension of TiO_2_ revealed that they were both larvicidal and adulticidal against *Bovicola ovis* (sheep louse) and *Hippobosca maculate* (hematophagous fly) [45]. The antioxidant and antibacterial properties of TiO_2_ nanoparticles, synthesized using an extract from *Psidium guajava*, were evaluated against *Pseudomonas aeruginosa, Staphylococcus aureus, Proteus mirabilis, Aeromonas hydrophila*, *E. coli*, and other pathogens. In addition, the antioxidant and antibacterial properties of TiO_2_ nanoparticles were evaluated in nanometer scale-up and in bulk [46].

### 3.5. Indium Oxide (In_2_O_3_), Iron Oxide, Lead, and Selenium Nanoparticles

Using a variety of plants, a number of new types of metal oxide and metal nanoparticles were biologically synthesized. Indium oxide nanoparticles were synthesized from aloe vera leaf extract (*aloe barbadensis Miller*). After primary synthesis, for the production of nanoparticles, the precipitates were thermally treated at 400–600 °C. The size of the synthesized spherical nanoparticles ranged from 5 to 50 nm and the size was dependent on the reaction temperature [47]. In a number of environmental remediation technologies, iron nanoparticles are very important. Thus, a number of studies have focused on green chemistry to synthesize these iron (Fe) nanoparticles. For instance, to synthesize Fe nanoparticles, sorghum bran aqueous extracts have been used. Recently, Pattanayak et al. [48] synthesized spherical iron nanoparticles that had particle sizes of 100 nm from *Azadirachta indica* (neem) leaf extract. A while ago, Shah et al. synthesized iron nanoparticles from different plant extracts, such as *Cymbopogon citratus* (lemon grass tea), *Datura innoxia, Tridax procumbens, Calotropis procera, Tinospora cordifolia*, and *Euphorbia milii*. Stem extract was used to synthesize the smallest spherical nanoparticles, from 13 nm, the widest size range (43–342 nm) of nanoparticles synthesized from *Cymbopogon citratus* leaf extract [49]. Lead (Pb) and selenium (Se) are two other significant nanoparticles that have been synthesized biologically. Joglekar et al. [50] were able to synthesize spherical Pb nanoparticles form *Jatropha curcas* latex, having sizes ranging from 10 to 12.5 nm. Lately, Sasidharan et al. [51] have been able to synthesize spherical selenium (Se) nanoparticles from citrus reticulata peel extract, with a size of 70 nm.

## 4. Green Synthesis of Metal Nanoparticles

**Figure 2:**
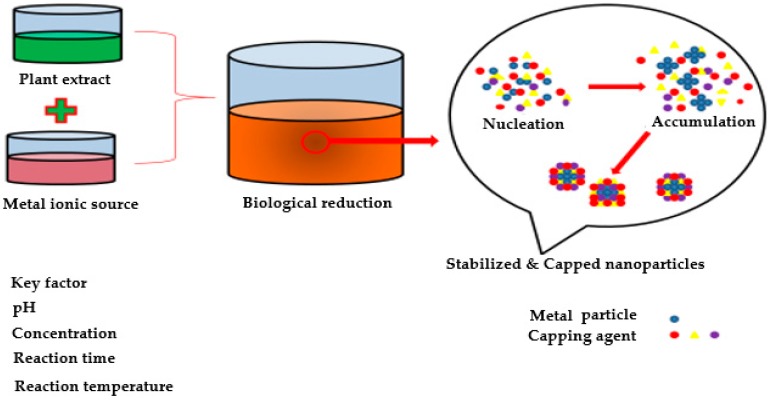
Biological synthesis of nanoparticles by using plant extracts.

For a long time, it has been known that plants have the potential for biological reduction of metallic ions and hyper-accumulation [52,53]. Because of such remarkable properties, plants have been considered a more environmentally friendly biological method for synthesis of metallic nanoparticles, and also useful for detoxification applications [54]. Plant extracts contain various bioactives, such as alkaloids, proteins, phenolic acids, sugars, terpenoids, and polyphenols, which have been found to have an important role in first reducing and then stabilizing the metallic ions, as shown in Figure 2. 

The shape and size of nanoparticles mainly depend on the variation in composition and concentration of active biomolecules of different plants, and their interaction with the aqueous metal ions. Especially in chemical and biological synthesis of nanoparticles, the aqueous metal ion precursors from metal salts are reduced, which results in colour change of the reaction mixture and provides a quantitative indication of nanoparticle formation. More importantly, the nanoparticles synthesized from reducing agents may show general toxicity, engendering serious concern for developing environmentally friendly processes. The process of the formation of nanoparticles begins by mixing a metal–salt solution with a sample of plant extract. During the synthesis of nanoparticles, biochemical reduction of the salt solution starts immediately and the change in colour of the reaction mixture indicates the formation of nanoparticles. During synthesis, initially there is an activation period process in which metal ions are converted to zero-valent state from their mono or divalent oxidation states, so that the nucleation of such reduced metal atoms takes place [55]. Furthermore, the process of nanoparticle synthesis is followed by the integration of smaller neighbouring particles to form larger nanoparticles, which are thermodynamically stable, and, subsequently, the metal ions are reduced biologically. In this way, growth progresses and nanoparticles aggregate to form a variety of shapes such as spheres, cubes, triangles, rods, wires, hexagons, and pentagons. In the final stage of the process, the ability of plant extract to stabilize the nanoparticle finally determines its stable morphology. Significantly, the quality, size, and morphology of the nanoparticles are influenced by properties of the plant extracts; mainly its concentration, reaction time, metal salt concentration, reaction solution pH, and temperature [56,57].

## 5. Green Nanotechnology: Risk Aspects

**Figure 3:**
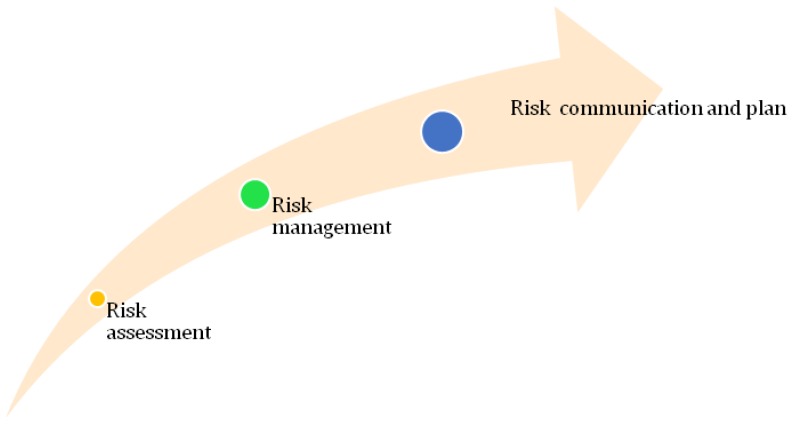
Risk aspects of green nanotechnology.

The invention of nanotechnology application in the field of green technology has raised concerns regarding the nanomaterial impact on health and safety of workers. This urgently requires scientific, technological, and governmental efforts to manage such kinds of risks for the workforce. This means detecting genuine risks derived from nanomaterial exposure in the workplace, that is, “risk assessment”: To plan the control measures of “risk management” and, finally, to communicate the plan. Overall, all these steps of risk aspects are critical and will be discussed in the following section, which aims to protect the worker from harm and provide all the benefits of green nanotechnology for society (Figure 3). 

## 6. Risk Assessment

Risk assessment of nanomaterials includes the same processing steps that are used in the risk assessment of other types of materials/chemicals [58]. These include hazard identification, hazard characterization, dose-response relationships, and assessment of exposure for the different scenarios. Unfortunately, the risk assessment process of nanomaterials still suffers from a deficiency of toxicological data for a variety of nanomaterials. Furthermore, the definition of vital health effects, such as genotoxicity, pulmonary toxicity, or carcinogenicity in conditions of long-term and low-dose exposure, are approaching realistic scenarios that require attention [59]. For the characterization of occupational nanomaterial risks, the exposure assessment remains a fundamental condition. Efforts should be made to overcome practical barriers that are related to the novelty of green-nanomaterial exposure scenarios, the variations in how to detect and classify nanomaterials, and the queries about the metrics for health-related sampling. That aside, it is also important to carry out biological monitoring studies, which are important to define possible biomarkers of nanomaterials exposure, and effects that are prospectively tested, validated and used in occupational risk evaluation [60].

## 7. Risk Management

The aim of risk assessment is to provide computable predictions of risks assisting their evidence-based management [61]. To effectively manage the potential risks related to green nanotechnology, a plan of risk management including a hierarchy of controls should be emphasized [62]. The first step of planning is to determine potential exposure to workers, measuring and identifying how this exposure may differ depending on the job task. After identification, potential worker exposure should be managed by using the hierarchy of controls that starts with hazard elimination, adopting a green chemistry by substitution with a non-hazard, and an introduction of engineering controls, including enclosed systems. Administrative control should follow these steps, including training programs by which companies communicate with workers to find out if they have the information to sufficiently understand the routes and nature of potential nanomaterial exposure in the workplace, adequate job procedures, possible risks, preventive and protective measures, and the policies adopted. In this context, it should be important to improve insufficient or inadequate information for workers present on safety data sheets. Risk management, including the use of personal protective equipment (PPE), including respiratory and eye protection, gloves, and lab coats, is the final step for exposure control.

## 8. Risk Communication

Risk communication is a crucial part of green nanotechnology, relating to the healthy origination and sustainable development of general public transparency. In terms of making available complex technical and health information, risk communication should be made effective, in language that should be accessible and understandable to the general population. Importantly, regulatory scientists, researchers, workforce representatives, industry, and governmental authorities should be engaged in a dialogical progressive communication of the potential green nanotechnology risks, with the objective to form adequate perceptions and attitudes. This is tremendously important to ensure that the spread as well as promotion by mass media involves appropriate information regarding the benefits and challenges of green nanotechnology, protecting public opinion from both unrealistic prospects and excessive consciousness in this regard.

## 9. Conclusions

As green nanotechnology becomes more commercialized, it will have the potential to become an industry with very strong green credentials. As a general conclusion, it can be said that green nanotechnology involves challenging work in the pharmaceutical industry. Ultimately, however, it improves the quality of life, promotes environmentally friendly commitments, as well as ethical values in the field of nanotechnology.

## Figures and Tables

**Table 1:** Herbal drug-loaded nanoparticles.

Formulation	Active Ingredients	Biological Activity	Method of Preparation	References
Curcuminoids solid lipid nanoparticles	Curcuminoids	Anticancer and antioxidant	Micro-emulsion technique	[8]
Glycyrrhizic acid loaded nanoparticles	Glycyrrhizin acid	Antihypertensive and anti-inflammatory	Rotary-evaporated film ultrasonication method	[9]
Nanoparticles of cuscuta chinensis	Flavonoids and lignans	Hepatoprotective and antioxidant effects	Nanosuspension method	[10]
Artemisinin nanocapsules	Artemisinin	Anticancer	Self-assembly procedure	[11]
Berberine-loaded nanoparticles	Berberine	Anticancer	Ionic gelation method	[12]
CPTencapsulated nanoparticles	Camptothecin	Anticancer	Dialysis method	[13]
Taxel-loaded nanoparticles	Taxel	Anticancer	Emulsion solvent evaporation	[14]

**Table 2:** A selection of nanoparticles synthesized by various plants.

Plant	Nanoparticle	Size (nm)	Shape	Reference
Aloe vera	Au & Ag	50 to 350	Spherical, triangular	[18]
Aloe vera	In_2_O_3_	5 to 50	Spherical	[19]
Citrullus colocynthis	Ag	31	Spherical	[20]
Curcuma longa	Pd	10 to 15	Spherical	[21]
Diopyros kaki	Pt	15 to 19	Crystalline	[22]
Eucalyptus macrocarpa	Au	20 to 100	Spherical, triangular, hexagonal	[23]
Mangifera indica	Ag	20	Spherical, triangular, hexagonal	[24]
Rhododendron dauricum	Ag	25 to 40	Spherical	[25]
Psidium guajava	Au	25 to 30	Spherical	[26]
Pyrus sp. (Pear fruit extract)	Au	200 to 500	Triangular, hexagonal	[27]
Terminalia catappa	Au	10 to 35	Spherical	[28]
